# Another place, another timer: Marine species and the rhythms of life

**DOI:** 10.1002/bies.201000096

**Published:** 2011-01-21

**Authors:** Kristin Tessmar-Raible, Florian Raible, Enrique Arboleda

**Affiliations:** Max F. Perutz Laboratories, University of ViennaVienna, Austria

**Keywords:** chronobiology, ecology, lunar periodicity, marine biology, photobiology

## Abstract

The marine ecosystem is governed by a multitude of environmental cycles, all of which are linked to the periodical recurrence of the sun or the moon. In accordance with these cycles, marine species exhibit a variety of biological rhythms, ranging from circadian and circatidal rhythms to circalunar and seasonal rhythms. However, our current molecular understanding of biological rhythms and clocks is largely restricted to solar-controlled circadian and seasonal rhythms in land model species. Here, we discuss the first molecular data emerging for circalunar and circatidal rhythms and present selected species suitable for further molecular analyses. We argue that a re-focus on marine species will be crucial to understand the principles, interactions and evolution of rhythms that govern a broad range of eukaryotes, including ourselves.

## Introduction

**Figure 1:**
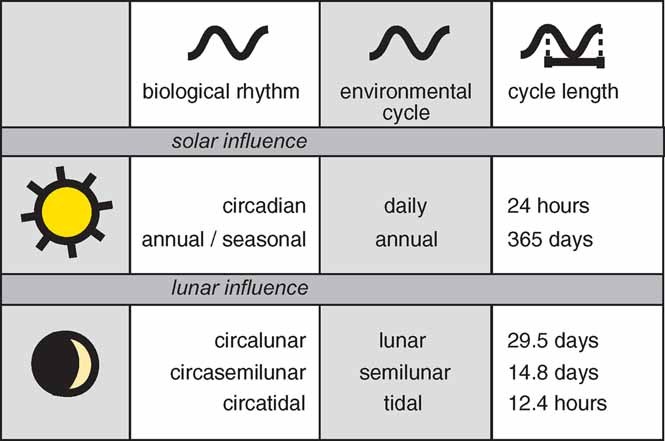
Common biological rhythms under solar and lunar influence. The overview lists a given biological rhythm (left), along with the corresponding environmental cycle (middle) and the periodicity of this environmental cycle (right). For simplicity, no distinction is made between the synodic (29.5 days) and the sidereal (27.3 days) lunar period. For additional terminology, see Box 1.

Life evolved in the sea, an ecosystem that is governed by a multitude of environmental cycles (Box 1). These include not only the daily day-night cycle, but also cycles with shorter or longer periods, such as tides, lunar/semi-lunar cycles or the seasons. Organisms in the marine environment have adapted to these steady cycles over millions of years, and use them for synchronization on many levels. Depending on the species, behaviour, reproduction, physiology or even cell divisions are tuned to environmental cycles with differing periods, resulting in a range of biological rhythms (Box 1). Periodic changes of external stimuli – such as light or pressure – provide cues for these rhythms. In all known cases, these zeitgeber (Box 1) stimuli are either directly or indirectly linked to the periodical recurrence of sun or the moon (Fig. 1). A number of molecular players have now been uncovered that explain how species receive zeitgeber stimuli, and what constitutes the underlying molecular networks. But to date, these molecular data almost exclusively cover the rhythms under solar control.

**Box 1**

**Concepts and terminology**

**Environmental cycle**: Periodically re-occurring natural conditions (e.g. day/night, high/low tides, moon phases, seasons).

**Biological rhythm**: Periodically re-occurring specific conditions within an organism (e.g. behavioural activity, metabolic state, gonadal maturity, etc.). Rhythms are typically in accordance with environmental cycles (see Fig. 1).

**Zeitgeber**: Environmental stimulus that serves as a synchronization cue.

**Molecular clock**: Molecular system that is able to maintain a given biological rhythm even under free-running conditions, i.e. in the absence of a zeitgeber. The molecularly best understood clock is the *circadian* clock. A unifying principle of eukaryotic circadian clocks is the existence of transcriptional/translational autoregulatory feedback loops 3.

**Entrainment**: Synchronization of a clock with the respective environmental cycle.

**Periodicity**: Cycles, rhythms and clocks are distinguished by their respective period length (common periodicities are listed in Fig. 1).

In contrast to their marine relatives, terrestrial model species, such as the fruitfly, the mouse or the thale cress, only display overt circadian and, in some instances, seasonal, rhythms. Therefore, our molecular understanding of these two types of rhythms is most advanced. A unifying principle of eukaryotic circadian rhythms is that they rely on negative transcriptional/translational feedback loops formed by a set of regulatory genes 1, 2. Importantly, this molecular system is able to maintain rhythmic circadian changes even under free-running conditions, i.e. in the absence of outside stimuli, and is therefore called circadian clock (Box 1). A number of studies now provide a detailed mechanistic understanding of how circadian clocks operate. This includes the role of protein turnover, specific post-translational modifications by de-actylation, methylation and phosphorylation, as well as the involvement of signalling cascades (reviewed in ref. 3).

**Figure 2:**
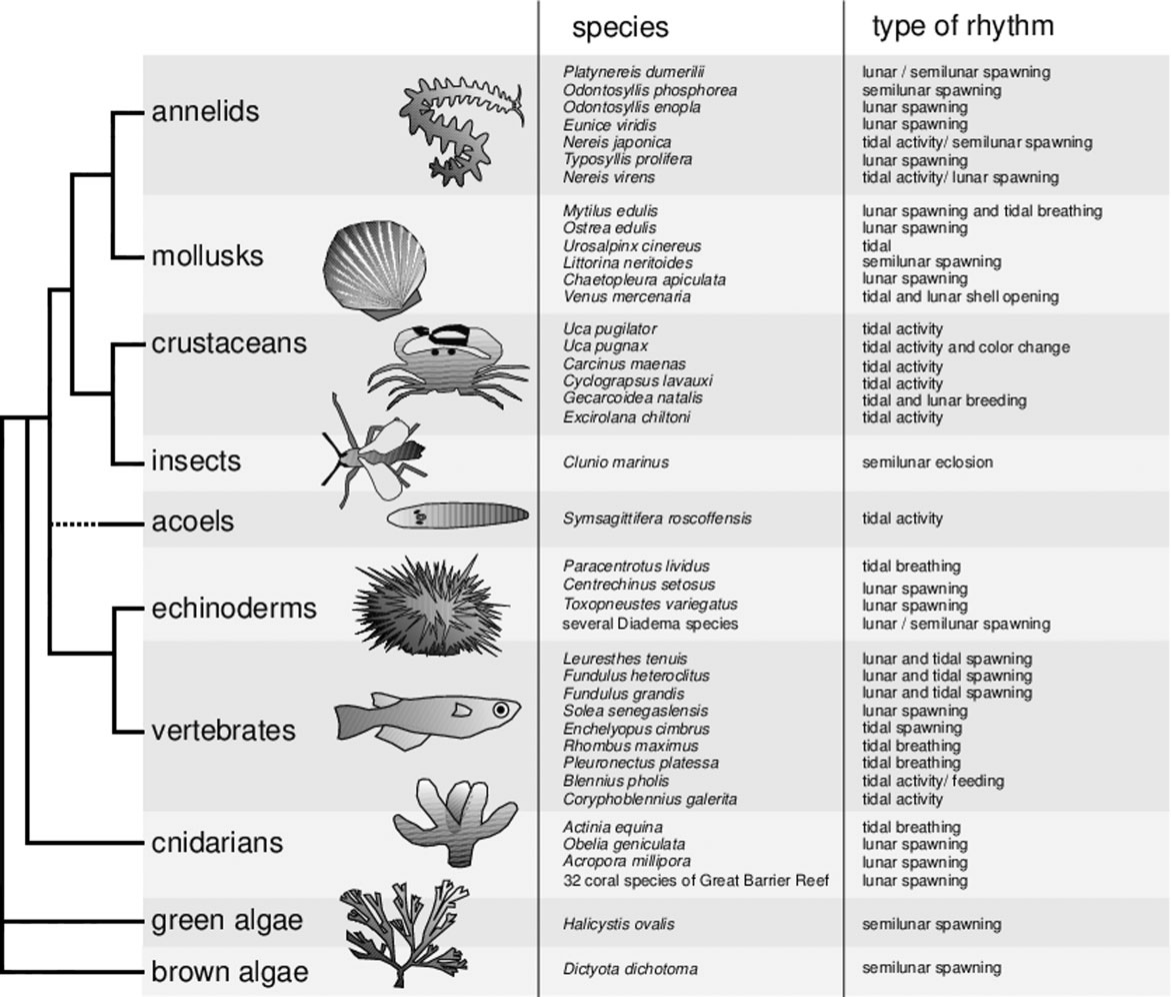
Lunar-controlled rhythms are widespread and of fundamental importance for marine organisms. Simplified phylogeny of metazoan groups with representatives exhibiting moon-controlled rhythms. In most mentioned cases evidence for a free-running lunar clock mechanism exist. Note that it is likely that all the mentioned species also possess a circadian clock and many also exhibit seasonality. All mentioned groups represent marine species. Lunar-controlled rhythms have also been described outside metazoans in green and brown algae (bottom) 9, 11–13, 58, 62, 86, 87.

However, free-running rhythms have not only been observed in the context of circadian clocks, but exist also for typical ‘marine rhythms’ of shorter and longer periods, like tidal or lunar rhythms (representative species listed in Fig. 2). This implies that the respective species possess multiple clock-like systems. Like their circadian counterparts, they allow species to anticipate important regular environmental changes; and they can do this even under circumstances when the environmental cues would be misleading (e.g. due to weather conditions).

In this article, we focus on the value of marine model species for molecular chronobiology. We first highlight important lunar rhythms, which are commonly found among marine species, but are far less, if at all, reliably described in terrestrial species. Next, we review how species adjusting to both lunar and solar cycles coordinate the underlying clocks, again a phenomenon only well known for marine species. Finally, we discuss the value of marine model species to shed light on the evolution and diversification of known molecular clocks.

Throughout the article, our main emphasis is on molecular aspects of ‘marine’ rhythms; for their behavioural or physiological dimensions, we refer the interested reader to recent overviews 1, 2, 4–6.

## Rhythms under lunar control

Historically, the remarkable discrepancy in our molecular understanding of solar and lunar rhythms can be traced back to the beginning of modern chronobiology. Biased by the availability of molecular model systems, molecular chronobiology has so far almost entirely focused on the analyses of a few selected land model species. Although nocturnal light can have slight effects on the daily rhythm of some land species 7, none of them display lunar-controlled rhythms. Consequently, no molecule has so far been clearly implicated in a moon-entrained clock. In the following sections, we present selected marine species that could serve as molecular models for two of the most common lunar-controlled rhythms – circatidal and circasemilunar/circalunar rhythms.

## Circatidal rhythms and clocks

Tides are caused by the gravitational forces of the sun and moon on the earth's water masses. Coastal areas can either exhibit semidiurnal tides (two high and two low waters per day) or diurnal tides (one high and one low water per day) where the former has a period of 12.4 h (i.e. half a lunar day), and the latter 24.8 h (i.e. a lunar day).

**Figure 3:**
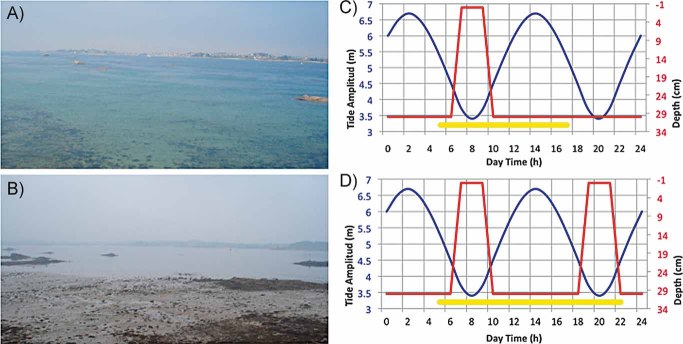
Habitat and rhythms of *S. roscoffensis*. **A-B:** Shallow coastal areas in Roscoff (France) during high and low tide periods, either covering or exposing the natural habitat of *S. roscoffensis*. **C:** During normal tide fluctuations over 24-hour periods (blue line), *S. roscoffensis* migrate to the surface of the sand or stay burrowed during low and high tides, respectively (red line). This process is dependent on the presence of sunlight (yellow line). **D:** In summer months, when long days overlap two low tide periods, *S. roscoffensis* migrate twice a day to expose themselves to sunlight. (C, D: after 88).

The rise and fall of water has a significant impact on the organisms living in the tidal zones. Humidity, salinity, temperature, oxygen levels, water current, sun irradiation, food availability, hydrostatic pressure and also predator exposure change due to the tides. Therefore, molecular clock mechanisms that allow a given species to anticipate the tidal change can be of critical importance to avoid exposure to life-threatening conditions. Indeed, the existence of circatidal clocks is evident from a number of species (Fig. 2). The first organism for which such a clock was described was the acoel flatworm *Symsagitiffera roscoffensis* 8–10 (Fig. 3; formerly in the genus *Convoluta*). Notwithstanding the uncertainty of the acoels' phyletic position (reviewed in ref. 11), their interest for chronobiology is undisputed, as they allow the circatidal clock to be molecularly assessed. EST and BAC sequences for the animal are forthcoming (P. Martinez and X. Bailly, personal communication).

*S. roscoffensis* live in groups of thousands of translucent individuals in shallow coastal areas in the southern part of the English Channel. The obligate endosymbiotic association with the microalga *Tetraselmis convolutae* dictates the tidal activity cycles of *S. roscoffensis*. During low tides, acoels emerge from the sand and expose themselves and their photosynthetic endosymbionts to the sunlight. Prior to the tide rising, acoels burrow again into the darkness of the sand. During the summer months, this migration cycle follows a circatidal rhythm of 12.4 h (Fig. 3). Experiments from the early 20th century showed that when adult animals were removed from their environment and placed in a glass container in the laboratory, they maintained free-running movement cycles for 5 days 8–10. This observation provides the first evidence for the existence of a circatidal clock in a marine organism.

Physiological and behavioural circatidal rhythms have been studied in a variety of other marine organisms, e.g. in sea spiders 12, crabs, crustaceans, sea anemones and various molluscs (see Fig. 2 and associated references). For most of those, it has also been shown that they are under the control of a tidal clock 13.

In the mussel *Mytilus californianus*, a first systematic study has been performed to identify molecular changes associated with the tidal cycle 14. Expression levels of cell division and metabolism genes appear to be anti-correlated in this species, suggesting that these cellular functions occur at separate time points, probably reflecting different favourable environmental conditions. Any clear association between the tidal cycle and gene expression levels, however, is missing, possibly owing to the low resolution of the study 14.

## Circalunar rhythms and clocks

Classical authors – starting with Aristotle – had already seen a connection between the different phases of the moon and the size of certain marine invertebrates. Zoological descriptions of the early and mid 20th century have since re-established the connection between the apparent size of marine animals with moon. More precisely, these studies have revealed that the maturation of gonads in these animals depends on a lunar cycle (see e.g. 15, 16 and Fig. 2). In species where the gonads contribute to a large proportion of the body mass, such as sea urchins, this effect is particularly prominent.

In contrast to the short period rhythms of the tides that most obviously manifest themselves in behavioural changes of animals, semi-lunar and lunar cycles are most prominently involved in reproductive events such as mass synchronous spawnings. This has been documented for a broad range of marine metazoans that include corals, sea urchins, bristle worms, molluscs and fish, as well as non-metazoans, such as green and brown algae (Fig. 2 and associated references). Lunar-controlled reproductive rhythms may be even further widespread than currently known, as in many species, the time of reproduction has not been regularly documented.

Typically, species reproducing with a circalunar or -semilunar rhythm rely on external fertilization, releasing the gonadal products of each individual into the open water. Whereas species with internal fertilization can tolerate differences in the maturation of each partner, successful fertilization in such broadcast spawners critically relies on tight synchronization between male and female maturation and the release of germ products. The regular cycle of the moon provides these organisms with a steady timeframe that can be used for synchronization even across widespread populations.

## Towards a molecular understanding of light-entrained circalunar rhythms and clocks

Coral reefs represent a habitat that is characterized by extensive lunar reproductive rhythmicity. In the Great Barrier Reef, more than 30 coral species display synchronous spawning in accordance with the lunar phase 17. Likewise, several coral reef fish species follow lunar reproductive rhythms (see below). One of the coral species that has been studied in more detail is *Acropora millipora*. It spawns once a year, at a defined lunar phase. A recent study suggests that an *Acropora* cryptochrome (Cry) molecule is influenced by the lunar rhythm 18. Members of the *cry* gene family are present throughout all kingdoms and play a crucial role in circadian rhythms 2, 19, 20. In mammals, vertebrate-type Crys act as transcriptional repressors that control the circadian clock 21, whereas the blue light/UV-A photoreceptor activity of Crys acts as input in the circadian clock of insects 22, 23 and plants 19.

*Acropora* harbours at least three different members of the *cry/phl* family, a *cry-dash, cry1* and *cry2* 18. Of those, the RNA and protein levels of Cry2 are affected by exposure to lunar light, leading the authors to suggest that Cry2 might be a lunar light sensor 18. The apparent changes in Cry levels, along with the evolutionary conservation of the molecule, make it an attractive candidate for a molecule involved in circalunar rhythms. However, it is not yet clear if *Acropora* Cry2 can actually act as a light receptor and hence mediate a lunar light stimulus.

Coral reef fishes represent another group in which circalunar rhythms have been described (reviewed in ref. 24). Among those, rabbitfish species display cycles of gonadal maturation and spawning in accordance with the lunar cycle. Molecular studies have shown that the observed histological changes accompanying gonad differentiation correlate with fluctuations of steroid hormones known to play a role in gonadal development 24–27.

Another set of observations has shown the influence of moonlight on components of the circadian clocks in rabbitfishes 28, 29. Melatonin acts as a major output molecule of the circadian clock in vertebrates, with levels typically peaking during the night. In *Siganus guttatus* and *Siganus canaliculatus*, plasma levels of melatonin have been found to be elevated in new moon nights compared to full moon nights. Similarly, pineal mRNA levels of *Siganus guttatus per2*, a likely core component of the circadian clock, differed significantly between full moon and new moon nights 30. Along with additional lunar changes in the mRNA levels of a candidate *S. guttatus* melatonin receptor (referred to in ref. 31), and measurements on cultured pineal tissue from the same species 32, these data suggest that lunar light is received by the rabbitfish pineal and thereby impacts on the circadian fluctuations of melatonin.

However, caution has to be used for the interpretation of these molecular studies. Also in *Drosophila*, which does not exhibit any biological circalunar rhythm, dim light of in the range of lunar light intensity can directly influence expression of circadian clock genes 7. Therefore, in the absence of free-running experiments, it remains unclear if the observed changes in levels of *Acropora* Cry2, *Siganus per2, melatonin receptor* or melatonin are caused by an endogenous lunar clock or directly result from illumination differences. Indeed, a study in soles suggests rather the latter to be the case 33. Furthermore, these observations do not necessarily imply that any of these molecules are functionally involved in a circalunar rhythm or clock.

Polychaetes (bristle worms) are a particularly attractive group to study lunar periodicity. Not only are the mass spawnings of polychaete species, such as the bioluminescent fire worm of Bermuda (*Odontosyllis*), or the famous Palolo worms in the Southern Pacific (*Palolo viridis*), spectacular and fascinating natural phenomena, in selected animals from this group, lunar reproductive periodicity also has a long history of scientific research 16, 34–41. Among these species is *Platynereis dumerilii* (Fig. 2), the first organism for which the entrainment to a lunar cycle was shown to only depend on nocturnal light stimuli 42–44. Classical studies, including one free-running experiment, indicate that *Platynereis* possesses an endogenous circalunar clock, and that lunar light acts as a zeitgeber for this clock 42. Notably, *Platynereis* has recently started to emerge as a novel molecular model system with large sequence resources and molecular tools 45–50. These resources, along with its ancestral-type nervous system 51–54, now make this organism an ideal starting point not only to unravel the molecular principles of its circalunar clock, but also to place it in an evolutionary context.

Another invertebrate that appears very promising for the molecular analyses of circalunar rhythms is the marine midge *Clunio marinus*. Similar to *Platynereis*, a wealth of classical studies attests to its circalunar rhythm of reproduction 55–58. *Clunio marinus* lives in the tidal zones of rocky beaches, spending most of its life cycle underneath the water. Only the few hours from eclosion to reproduction need to take place outside the water. At the end of this period, females deposit their eggs on algae that only emerge from the water during spring tides, and are otherwise covered by water 59. In accordance with the critical importance of this final stage of their life cycle, *Clunio* displays a strong preference to hatch and reproduce just during the spring tides, when the low water levels reach their extremes. The synchronization of these midges therefore does not primarily affect gonadal maturation, as is the case of the bristle worm, but the time point of their eclosion.

Similar to *Platynereis*, but in contrast to many other marine organisms, *Clunio* can be easily grown in an in-land laboratory. Free-running experiments indicate that *Clunio* possesses a circalunar clock that is able to anticipate the correct time window for eclosion 56. Importantly, naturally occurring *Clunio* strains display different hatching cycles that are precisely adapted to the tidal times in the different coastal areas from which the strains are isolated. Their comparison reveals that they only evolved in the relatively short time period after the last ice age 60. This makes *Clunio* a highly attractive model for population genetics to unravel the molecular players that determine differences of hatching peaks of different strains.

## How are different rhythms and clocks interconnected?

Projecting beyond the molecular mechanisms of single circadian, circalunar, circatidal or seasonal/circaannual rhythms, the next level of functional and evolutionary understanding will be to discern how these different types of rhythms are molecularly connected, and to what extent underlying molecular clocks share the same components. Interconnections between different rhythms are evident from many examples (Figs. 2 and 3 and 13, 61, 62). For instance, in species like *Clunio*, the light-sensory system involved in the entrainment of the circalunar clock is controlled in a circadian manner. This specific regulation of the sensory system offers an attractive explanation for how the animals can discriminate nocturnal light from daylight, and hence recognize the correct moon phases in the absence of obvious differences, e.g. in spectral composition of sun- and moonlight 58. Furthermore, lunar reproduction can be modulated by seasonal factors, restricting reproduction to only few reproductive months 16, 63. Likewise, most species that show lunar reproduction also have a defined circadian time window in which reproduction occurs. In exemplary cases, this interconnection allows precise timing of reproduction to specific hours of specific days of the reproductive month(s) 13, 14, 41, 64, 65. These intricate connections between different types of rhythms argue for the existence of common molecular regulators, for instance hormones or signalling metabolites, which mediate crosstalk between different molecular processes or even clocks within the same species.

The connections between different clocks have been intensively debated for circatidal and circadian rhythms in intertidal crabs. Two main hypotheses have been put forward to explain the circatidal locomotor activity of these crabs and their performance under constant laboratory conditions. The first hypothesis, the ‘circatidal/circadian clock model’, was developed by Naylor 66, supported by experiments showing a decoupling of circadian and circatidal rhythmicity in crabs 67. In essence, it postulates that locomotor activity is promoted by a circadian oscillator (24.8 h) and suppressed by an overlapping circatidal oscillator (12.4 h) (reviewed in refs. 68, 69). In the crab *Carcinus maenas*, the circadian oscillator would modulate the activity levels and by this determine that night activity is generally higher than day activity. The circatidal clock would suppress this general activity, leading to activity bursts (of different height) every 12.4 hours.

The second hypothesis, the ‘circalunidian-clock model’ originates from Palmer and associates in the late 80s (reviewed in ref. 70). It rejects the idea of a circatidal clock, instead suggesting two circalunar clocks at a period of 24.8 hours. Normally, these clocks would run in 180° antiphase to each other, generating activity bursts of the animals every 12.4 hours. Changes in the period length of either one of the two circalunar clocks would entail irregularities in the 12.4 hour cycles (such as a drift or splitting of the activity peaks) that occur under constant conditions in species like *Uca pugnax* or *Helice crassa* and are hard to reconcile with the circatidal/circadian clock model. The prediction is that in the absence of external stimuli, the coupling of both clocks can get lost. This provides a relative straightforward explanation how circatidal locomotor activity peaks under constant conditions can drift or temporarily disappear, a frequently observed phenomenon.

Supporting evidence for both models has been collected over the years (summarized in refs. 5, 6). In the polychaete *Nereis virens*, the acquired data fit both models 62, amphipod researchers have favoured the circatidal/circadian clock model 71, while for other organisms like the horseshoe crab and intertidal crabs data support the circalunidian-clock model 72, 73. Both models provide testable hypotheses for the underlying molecular machinery. It is thus likely that molecular research in those species will soon discriminate between both scenarios.

## Evolution and diversification of ‘marine’ rhythms and clocks

Given that the marine biosphere is the ancient environment in which life, with all its rhythms, evolved, marine species can provide crucial input on the origin and diversification of rhythms and their underlying clocks. This starts with the sampling of species used for chronobiological research. In light of the diverse tree of eukaryote phylogeny 74, it is obvious that the current molecular chronobiological models only represent small and specialized subsets of species. The study of other, molecularly unexplored groups is therefore promising to reveal additional strategies to cope with periodic environmental changes, and also delineate more precisely which aspects of the extant clocks are of ancient origin.

This principle can be well exemplified for the circadian clock. Within the eumetazoans, work on organisms other than *Drosophila* and mouse, such as honeybees, butterflies and sea urchins has already led to the novel insight that ancient Bilateria had clock molecules that were previously believed to be only present in either *Drosophila* or vertebrates, such as vertebrate-type and *Drosophila*-type *cryptochromes* (*crys*), *timeless* and *timeout* 75, 76. These findings have two implications: First, the eumetazoan ancestor (most likely a marine species) possessed a complex repertoire of circadian clock genes. Second, the current animal models use secondary simplifications of this ancestral circadian clock machinery.

The comparison between clock systems is not only informative in animals. Photosynthetic algae reveal details of the evolution of circadian clocks in plants. Research in the marine unicellular green alga *Ostreococcus* has served to reveal ancient aspects of the *Arabidopsis* circadian clock. Orthologs of two *Arabidopsis* core clock genes, timing of cab expression1 (TOC1) and circadian clock associated1 (CCA1), also play a central role in the alga. In contrast, the minimalistic genome of *Ostreococcus*, a picoeukaryote with only 13 Mbp of sequence, lacks other components of the *Arabidopsis* circadian clock, and therefore allows a core set of clock genes to be studied in a simpler context 77.

Marine species also provide insight into ancient correlations between clocks and the physiology of species. For instance, researchers studying regenerating mouse liver uncovered an unexpected link between the entry of cells into the G2 cell cycle phase and the circadian clock (reviewed in ref. 78). This finding might be less surprising in light of the fact that unicellular marine organisms, such as diatoms or green algae, synchronize their cell divisions in a circadian manner 79, 80. This coordination ensures harmonization with the algae's photosynthetic activity. The need to coordinate cell division with the organism's physiology might well represent an ancient marine condition still maintained in today's vertebrate liver cells. Similarly, in the diatom *Phaeodactylum tricornutum*, a single Cryptochrome molecule combines DNA repair function, light perception and circadian regulation. This Cryptochrome therefore appears to provide a missing link between the different functions of Cryptochromes in more diversified organisms 81.

Another example where ‘marine rhythms’ provide interesting physiological connections is the coupling of lunar reproduction and regeneration in several polychaetes. Especially in nereidids, the ability to regenerate is often anti-correlated with the production of eggs or sperm 82–85. This anti-correlation probably reflects the economy of energy expenditure in an organism with extreme fecundity, but it might also yield general insight into the mechanisms by which regenerative ability is restricted in other species, including vertebrates.

In summary, these examples attest to the value of marine species for understanding how molecular rhythms and clocks evolved and diversified along different evolutionary lineages. Moreover, they indicate that selected marine species have a strong potential to reveal new insights into the crosstalks between molecular clocks and physiological or cell biological events. With the advent of new molecular insight into marine non-circadian rhythms, it is likely that we will also learn more about the correlates or remnants of these rhythms in terrestrial models. Is it possibly more than sheer coincidence that the female reproductive cycle in humans lasts around a lunar month, or could this instead reflect some regulatory left-over from our evolutionary past? In this sense, the study of rhythms in our marine relatives can shed more light onto our own evolutionary past.

Whereas we have mostly focused on the interest of ‘marine rhythms’ for chronobiology, their molecular dissection will also advance studies in other areas of marine biology: Molecules involved in ‘marine rhythms’ will be useful to measure the effects of climate change or light pollution on the marine ecosystem and guide the search for improvement, e.g. by limiting light pollution to specific wavelengths or phases of the night. Finally, a better molecular knowledge on marine spawning rhythms could help to improve aquaculture and re-growth of species like corals.
